# Lasting enhancements in neural efficiency by multi-session transcranial direct current stimulation during working memory training

**DOI:** 10.1038/s41539-023-00200-y

**Published:** 2023-11-02

**Authors:** Yufeng Ke, Shuang Liu, Long Chen, Xiashuang Wang, Dong Ming

**Affiliations:** 1 Academy of Medical Engineering and Translational Medicine, Tianjin International Joint Research Centre for Neural Engineering, and Tianjin Key Laboratory of Brain Science and Neural Engineering, Tianjin University, Tianjin, PR China; 2 Haihe Laboratory of Brain-computer Interaction and Human-machine Integration, Tianjin, PR China; 3 The Second Academy of China Aerospace Science and Industry Corporation, Beijing, PR China

**Keywords:** Working memory, Human behaviour, Cognitive control

## Abstract

The neural basis for long-term behavioral improvements resulting from multi-session transcranial direct current stimulation (tDCS) combined with working memory training (WMT) remains unclear. In this study, we used task-related electroencephalography (EEG) measures to investigate the lasting neurophysiological effects of anodal high-definition (HD)-tDCS applied over the left dorsolateral prefrontal cortex (dlPFC) during a challenging WMT. Thirty-four healthy young adults were randomized to sham or active tDCS groups and underwent ten 30-minute training sessions over ten consecutive days, preceded by a pre-test and followed by post-tests performed one day and three weeks after the last session, respectively, by performing high-load WM tasks along with EEG recording. Multi-session HD-tDCS significantly enhanced the behavioral benefits of WMT. Compared to the sham group, the active group showed facilitated increases in theta, alpha, beta, and gamma task-related oscillations at the end of training and significantly increased P300 response 3 weeks post-training. Our findings suggest that applying anodal tDCS over the left dlPFC during multi-session WMT can enhance the behavioral benefits of WMT and facilitate sustained improvements in WM-related neural efficiency.

## Introduction

As a cognitive enhancement technique, transcranial direct current stimulation (tDCS) has been extensively studied over the past two decades, demonstrating its ability to improve working memory (WM) in both healthy and cognitively impaired subjects based on its ability to modulate neuronal excitability and plasticity^1–3^. Significant benefits of tDCS have been demonstrated by evidence that the performance benefits of the combination of tDCS and multi-day WM training (WMT) can persist over the long term, i.e., from one month to almost one year after the end of training^4–13^, and that these benefits have the potential to transfer to other untrained WM tasks^7,8,12,14–16^, fluid intelligence^17^ and even everyday tasks^9^. However, quantifying the effectiveness of tDCS+WMT in most previous studies has focused only on behavioral measures such as accuracy or reaction time (RT), which can provide a general but superficial understanding of cognitive effects. The lack of neurophysiological evidence makes it difficult to gain insight into the role of tDCS+WMT in the human brain.

Electroencephalography (EEG) allows for a non-invasive and temporally precise assessment of the neuromodulatory effects of WMT and brain stimulation. As measures of task-related brain dynamics, event-related potentials (ERPs) and event-related desynchronization/synchronization (ERD/ERS) can reflect cortical activation/deactivation^18^ and have been associated with WM performance fluctuations and neural efficiency^19,20^. For instance, theta oscillations have been critical for the maintenance of information in WM^21^, and frontal theta ERS enhancements have been associated with better WM performance^22,23^. As an indicator of cortical activation/deactivation, ERD/ERS has been used to measure neural efficiency, which is defined as the amount of neural resources needed to be engaged to solve a problem^24^. In addition to the ERD/ERS phenomenon, the extensively studied P300 response has been robustly linked to the intensity of attentional processing during retrieval of an item stored in WM^25^. P300 amplitude typically decreases with increasing WM difficulty because increasing WM load reduces the available attentional resources required to retrieve an item from WM^25,26^. These findings demonstrate that task-related ERD/ERS and P300 responses could characterize variations in the processing efficiency of neural circuits related to WM, thus providing promising approaches for investigating the effects of tDCS on WM performance.

The temporary or short-term neurophysiological effects of transcranial electrical stimulation (TES) have been substantially investigated in single-session studies using EEG measures^27–31^. Among the few multi-session WMT studies that have examined the effects of tDCS on EEG dynamics, modulatory effects on alpha oscillation have been considered an indicator of cumulative changes in cortical excitability and efficiency^32–34^. These findings have suggested that the neural efficiency of the neurocircuitry supporting WM processing may be enhanced by the application of tDCS. However, there is still insufficient convincing evidence that tDCS can modulate task-related brain activity and promote long-lasting improvements in neural efficiency. Some studies also failed to find significant modulatory effects of tDCS on task-related oscillations or ERPs in single-session studies^35^ and cognitive training studies^36–38^.

More importantly, few studies have examined the aftereffects of tDCS on these neurophysiological measures immediately after the end of WMT, typically at the end^36,39^ or one day after the last training session^4,32–34^. A recent study utilizing resting-state functional magnetic resonance imaging (fMRI) found improved brain network processing efficiency with 3 days of tDCS+WMT one day after training^40^. However, no significant effects of tDCS on brain activity were found in a 3-day tDCS+WMT study that quantified long-term neurobehavioral effects using ERPs one month after training^38^. Therefore, it remains an open question whether the cumulative effects of tDCS during WMT can produce lasting effects on the functional reorganization of cortical neural activity, which can be revealed by task-related EEG measures long-term after the end of training.

**Fig. 1:**
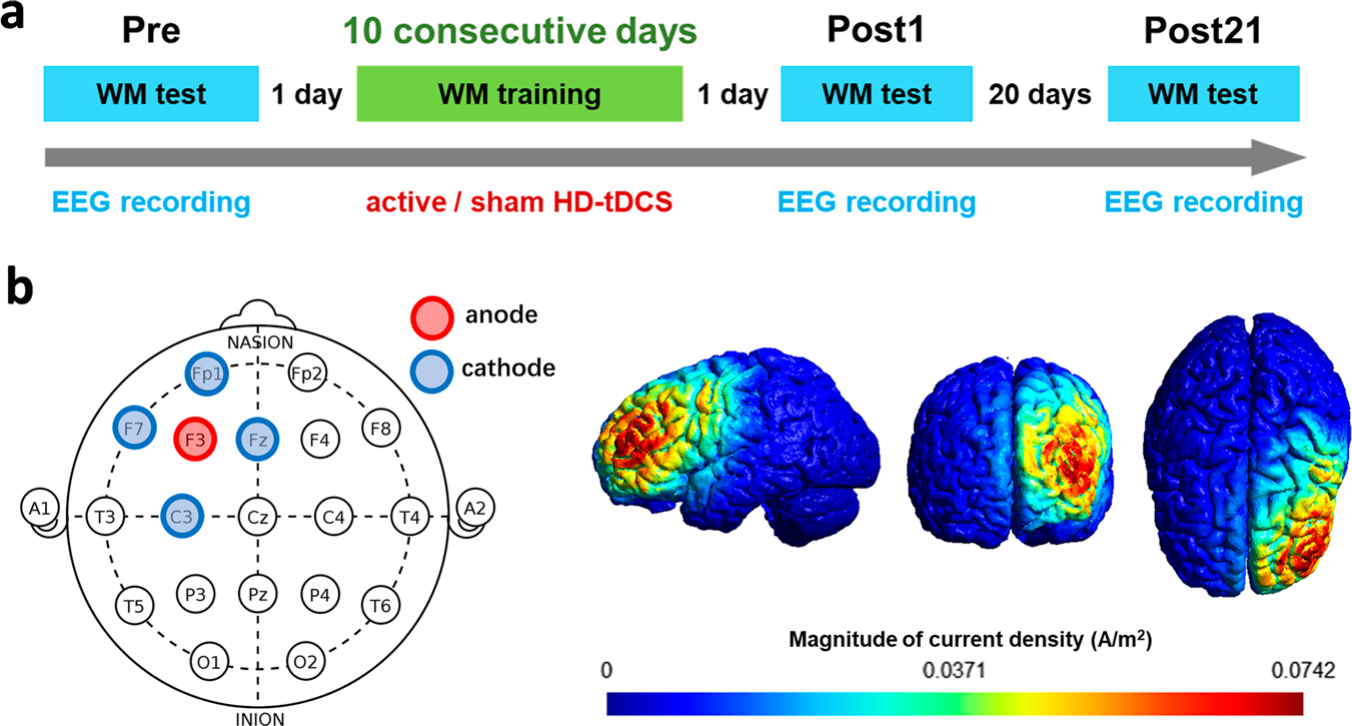
Details of experimental design. **a** Schematic of the experimental procedure. **b** Locations of the HD-tDCS electrodes and the corresponding simulation results of the magnitude of the current density (A/m²).

Given the paucity of research on the lasting aftereffects of tDCS on neurophysiological outcomes, this study sought to investigate changes in WM-related EEG dynamics immediately and relatively long term after the end of tDCS+WMT. A high-definition tDCS (HD-tDCS) montage, which can lead to focal activation of specific cortical areas^41^ and produce more robust changes in cortical reactivity^42^ compared to conventional bipolar tDCS, was applied over the left dlPFC during a consecutive 10-day challenging WMT (see Fig. 1 and the Methods section for details). WM performance was tested along with EEG recording before the start of the MWT (Pre-test) and one day (Post1-test) and three weeks (Post21-test) after the end of the WMT. Pre- and post-test performance was compared between the active and sham groups to examine the cognitive effects of HD-tDCS. More importantly, task-related EEG power and P300 responses, which may reflect the neural efficiency of WM processing, were analyzed to better understand the neurophysiological effects modulated by tDCS+WMT. We hypothesized that behavioral and neural effects of tDCS would be found not only at the Post1 test but also at the Post21 test, i.e., lasting neurobehavioral effects of tDCS were expected to be revealed after a relatively long time by examining the changes in task-related EEG power and P300 measures.

## Results

### Changes in WM performance of verbal 4- and 6-back tasks after training

**Fig. 2:**
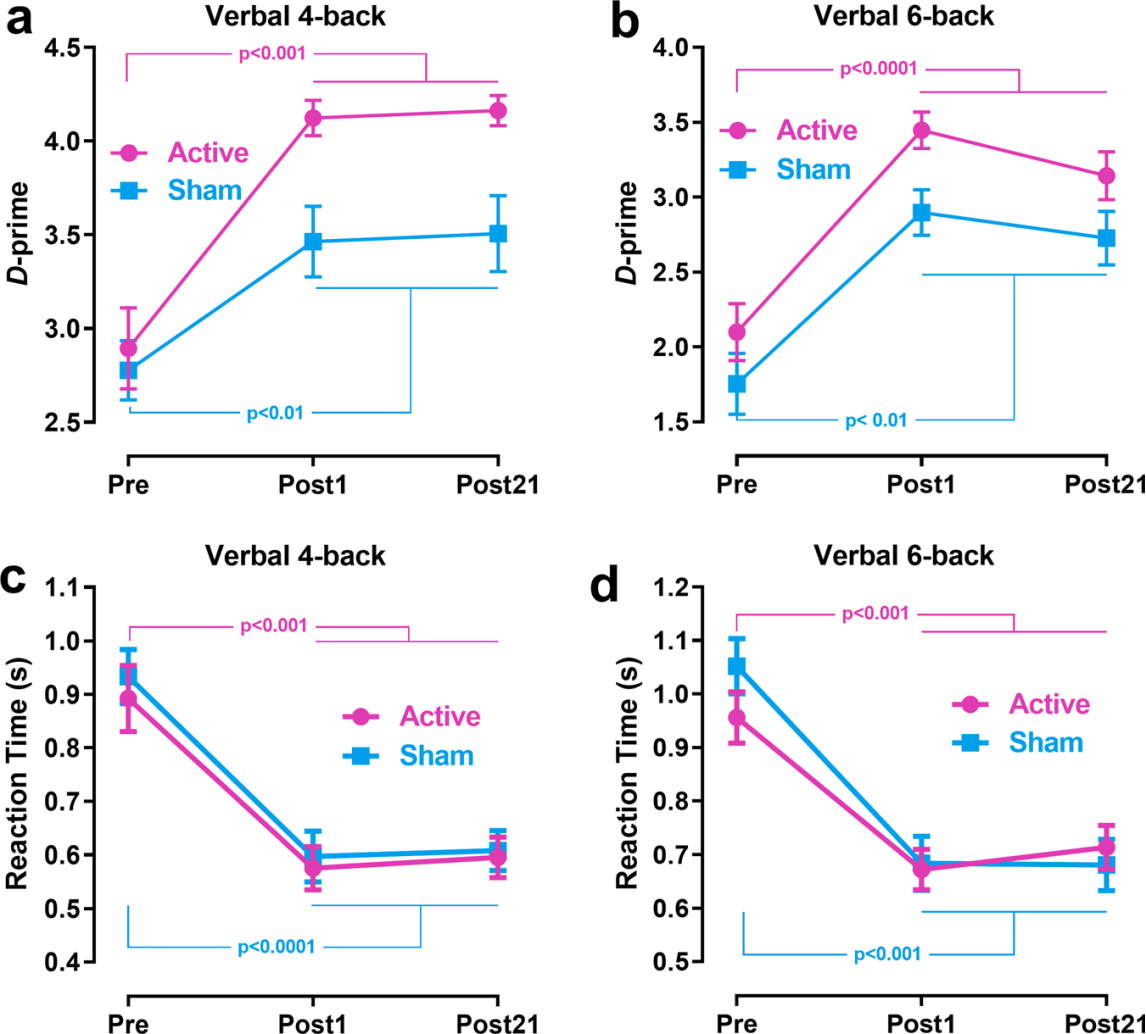
Mean WM performance (*d*-prime and reaction time). **a** and **b** show d-primes for verbal 4- and 6-back tasks, respectively. **c** and **d** show reaction times for verbal 4- and 6-back tasks, respectively. *P*-values were determined by Wilcoxon signed-rank tests. Error bars depict SEM.

The first goal of our study was to examine whether participants who received active tDCS during the ten days of WM training showed higher levels of WM performance compared to the sham group after the same time interval. Two-way mixed ANCOVAs were conducted to examine group and time effects on WM performance measures in 4-back and 6-back, respectively, controlling for the corresponding baseline measures (Pre-test) as covariates. Figure 2 reports the group average *d*-primes and RTs. The results of mixed ANCOVAs related to the effects of Pre-test, the main effects of group and time, and group-by-time interaction effects on *d*-primes and RTs are reported in Table 1. The unadjusted and baseline-adjusted descriptive statistics for *d*-primes and RTs at Post-tests are shown in Supplementary Table 1.

**Table 1:** Results of mixed ANCOVAs related to the effect of Pre-test, the main effects of group and time and group by time interaction effect on behavioral and EEG measures in verbal 4- and 6-back tasks.

Behavioral/EEG measure		Pre-test			Group			Time			Group-by-time interaction		
		*F*(1, 31)	*p*	${\eta }_{p}^{2}$	*F*(1, 31)	*p*	${\eta }_{p}^{2}$	*F*(1, 31)	*p*	${\eta }_{p}^{2}$	*F*(1, 31)	*p*	${\eta }_{p}^{2}$
Verbal 4-back	D-prime **#**	3.294	0.079	0.096	**10.932**	**0.002**	**0.261**	0.120	0.732	0.004	0.020	0.621	0.000
	RT **#**	**21.598**	**<0.0001**	**0.411**	0.004	0.953	0.000	1.466	0.235	0.045	0.000	0.975	0.000
	Theta power **#**	**5.181**	**0.030**	**0.152**	0.005	0.943	0.000	0.000	0.992	0.000	2.627	0.116	0.083
	Alpha power	**14.988**	**<0.001**	**0.341**	0.199	0.659	0.007	0.000	0.982	0.000	**11.112**	**0.002**	**0.277**
	Beta power	**7.553**	**0.010**	**0.207**	3.773	0.062	0.115	2.092	0.159	0.067	3.538	0.070	0.109
	Gamma power	**10.801**	**0.003**	**0.271**	**6.151**	**0.019**	**0.175**	2.430	0.130	0.077	0.739	0.397	0.025
	P300 GFP	**16.354**	**<0.001**	**0.361**	3.036	0.092	0.095	1.481	0.233	0.049	**4.168**	**0.050**	**0.126**
Verbal 6-back	D-prime	1.366	0.251	0.042	**4.511**	**0.042**	**0.127**	2.667	0.113	0.079	0.737	0.397	0.023
	RT **#**	**16.354**	**<0.001**	**0.345**	2.176	0.150	0.066	4.549	0.041	0.128	1.077	0.307	0.034
	Theta power	**10.691**	**0.003**	**0.269**	**5.323**	**0.028**	**0.155**	2.279	0.142	0.073	2.888	0.100	0.091
	Alpha power	**28.990**	**<0.00001**	**0.500**	**4.501**	**0.043**	**0.134**	**5.185**	**0.030**	**0.152**	**8.694**	**0.006**	**0.231**
	Beta power	**11.813**	**0.002**	**0.289**	**11.460**	**0.002**	**0.283**	3.646	0.066	0.112	1.924	0.176	0.062
	Gamma power	**16.603**	**<0.0004**	**0.364**	**13.987**	**<0.0009**	**0.325**	3.387	0.076	0.105	**8.402**	**0.007**	**0.225**
	P300 GFP #	**17.509**	**<0.001**	**0.376**	3.884	0.058	0.118	**6.053**	**0.020**	**0.173**	1.132	0.296	0.038

Bold indicates statistically significant results (*p* < 0.05). # Box-cox transformation was applied before ANCOVA due to normality violation.

#### D-prime

No significant effects of the Pre-test *d*-primes on the Post-tests were observed in both tasks. The main effects of group were significant, while the main effects of time and the group-by-time interaction effects in both tasks were non-significant after controlling for the Pre-test *d*-primes, indicating that the active group outperformed the sham group at the Post-tests. Wilcoxon signed-rank tests confirmed that participants’ *d*-primes increased significantly from Pre- to Post1- and Post21-tests for both tasks in both active and sham groups (Table 2). While group differences in baseline *d*-primes of both tasks were insignificant by applying Mann-Whitney tests [4-back: M-W = 159.500, *p* = 0.617, Rank-Biserial Correlation = 0.104; 6-back: M-W = 177.000, *p* = 0.274, Rank-Biserial Correlation = 0.225], these group differences were significant after training with higher levels of *d*-primes in the active group, compared to the sham group. Given that the two groups displayed the same level of performance in *d*-prime prior to training, it is less likely that higher levels of *d*-prime at Post-tests in the active group are associated with “more room to improve.” As indicated by the insignificant time effect, the insignificant changes from Post1- to Post21-test suggest that the WMT benefit may last three weeks after the training ended for both groups. Together, these results demonstrate that participants who received active tDCS showed lasting greater improvements in WM ability than those who received sham stimulation during the ten days of WMT.

**Table 2:** Results of Wilcoxon signed-rank tests for comparisons between Pre-test and Post-tests for behavioral and EEG measures in verbal 4- and 6-back tasks.

Behavioral/EEG measure		Post1 vs. Pre			Post21 vs. Pre		
		*z*	*p*	*rbc*	*z*	*p*	*rbc*
Active group, Verbal 4-back	D-prime	**3.527**	**<0.0001**	**0.974**	**3.516**	**<0.001**	**1.000**
	RT	**−3.456**	**<0.001**	**−0.954**	**−3.574**	**<0.0001**	**−0.987**
	Theta power	1.810	0.074	0.515	1.293	0.211	0.368
	Alpha power	2.430	0.013	0.691	1.344	0.193	0.382
	Beta power	**2.741**	**0.004**	**0.779**	**2.896**	**0.002**	**0.824**
	Gamma power	1.706	0.093	0.485	**2.585**	**0.008**	**0.735**
	P300 GFP	2.430	0.013	0.691	**3.516**	**<0.0001**	**1.000**
Sham group, Verbal 4-back	D-prime	**2.722**	**0.005**	**0.752**	**2.769**	**0.004**	**0.765**
	RT	**−3.574**	**<0.0001**	**−0.987**	**−3.574**	**<0.0001**	**−0.987**
	Theta power	2.172	0.029	0.618	**2.792**	**0.003**	**0.794**
	Alpha power	1.810	0.074	0.500	**3.051**	**0.001**	**0.868**
	Beta power	1.034	0.323	0.294	**3.103**	**<0.001**	**0.882**
	Gamma power	−0.982	0.348	−0.279	1.138	0.274	0.324
	P300 GFP	1.862	0.065	0.529	1.396	0.175	0.397
Active group, Verbal 6-back	D-prime	**3.621**	**<0.0001**	**1.000**	**3.621**	**<0.0001**	**1.000**
	RT	**−3.574**	**<0.0001**	**−0.987**	**−3.432**	**<0.001**	**−0.948**
	Theta power	**2.999**	**0.001**	**0.853**	**3.309**	**<0.001**	**0.941**
	Alpha power	**3.051**	**0.001**	**0.868**	**2.896**	**0.002**	**0.824**
	Beta power	**2.999**	**0.001**	**0.853**	**3.206**	**<0.0001**	**0.912**
	Gamma power	1.862	0.065	0.529	**3.258**	**<0.001**	**0.926**
	P300 GFP	1.655	0.105	0.471	**3.464**	**<0.0001**	**0.985**
Sham group, Verbal 6-back	D-prime	**3.053**	**0.001**	**0.843**	**2.817**	**0.003**	**0.778**
	RT	**−3.621**	**<0.0001**	**−1.000**	**−3.432**	**<0.001**	**−0.948**
	Theta power	0.621	0.562	0.176	2.327	0.018	0.662
	Alpha power	−0.569	0.597	−0.162	**2.844**	**0.003**	**0.809**
	Beta power	0.052	0.980	0.015	1.241	0.231	0.353
	Gamma power	−0.931	0.375	−0.265	−1.913	0.058	−0.544
	P300 GFP	0.207	0.860	0.059	0.776	0.464	0.221

Bold indicates statistically significant results (*p* < 0.0125).

*rbc* Ranked-Biserial Correlation.

#### RT

There were significant effects of the Pre-test RTs on RTs of the Post-tests. After controlling for the baseline RTs, the main effects of group, time, and group-by-time interaction effects were insignificant in both tasks. Wilcoxon signed-rank tests confirmed that participants’ RTs decreased significantly from Pre- to Post1- and Post21-tests in both active and sham groups (Table 2). Group differences in RTs of both tasks were not significant at Pre-test by applying Mann-Whitney tests [4-back: M-W = 129.000, *p* = 0.610, Rank-Biserial Correlation = −0.107; 6-back: M-W = 111.000, *p* = 0.259, Rank-Biserial Correlation = −0.232]. Together, these results demonstrate that participants’ RTs were not affected by tDCS intervention but decreased in responding to WMT. Moreover, the improvements in the RTs were maintained after three weeks.

### Changes in task-related EEG dynamics

We examined whether and how tDCS affected the possible WMT-induced changes in task-related EEG dynamics by conducting mixed ANCOVAs on the task-related EEG power and the P300 Global field power (GFP) measures at Post-tests in 4-back and 6-back, respectively, controlling for the corresponding Pre-test measures as covariates (see Methods). The results of mixed ANCOVAs related to the effects of the Pre-test, the main effects of group and time, and group by time interaction effects on ERD/ERS and P300 GFP measures are reported in Table 1. The unadjusted and baseline-adjusted descriptive statistics of these EEG dynamics at Post-tests are shown in Supplementary Tables 2, 3, and 4.

### Task-related EEG power

**Fig. 3:**
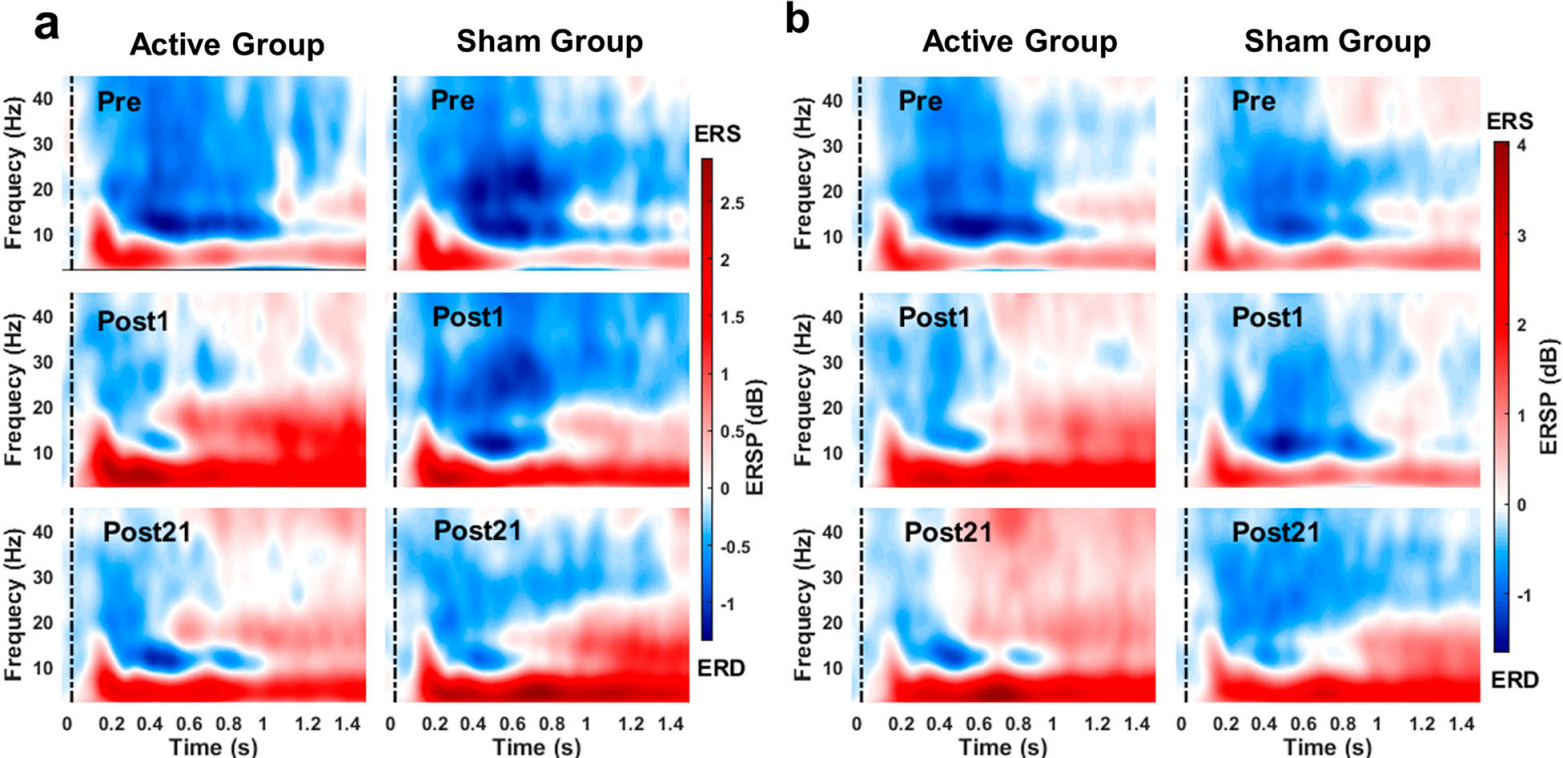
Grand-average time-frequency distributions of the frontal task-related EEG power. **a** verbal 4-back task. **b** verbal 6-back task.

**Fig. 4:**
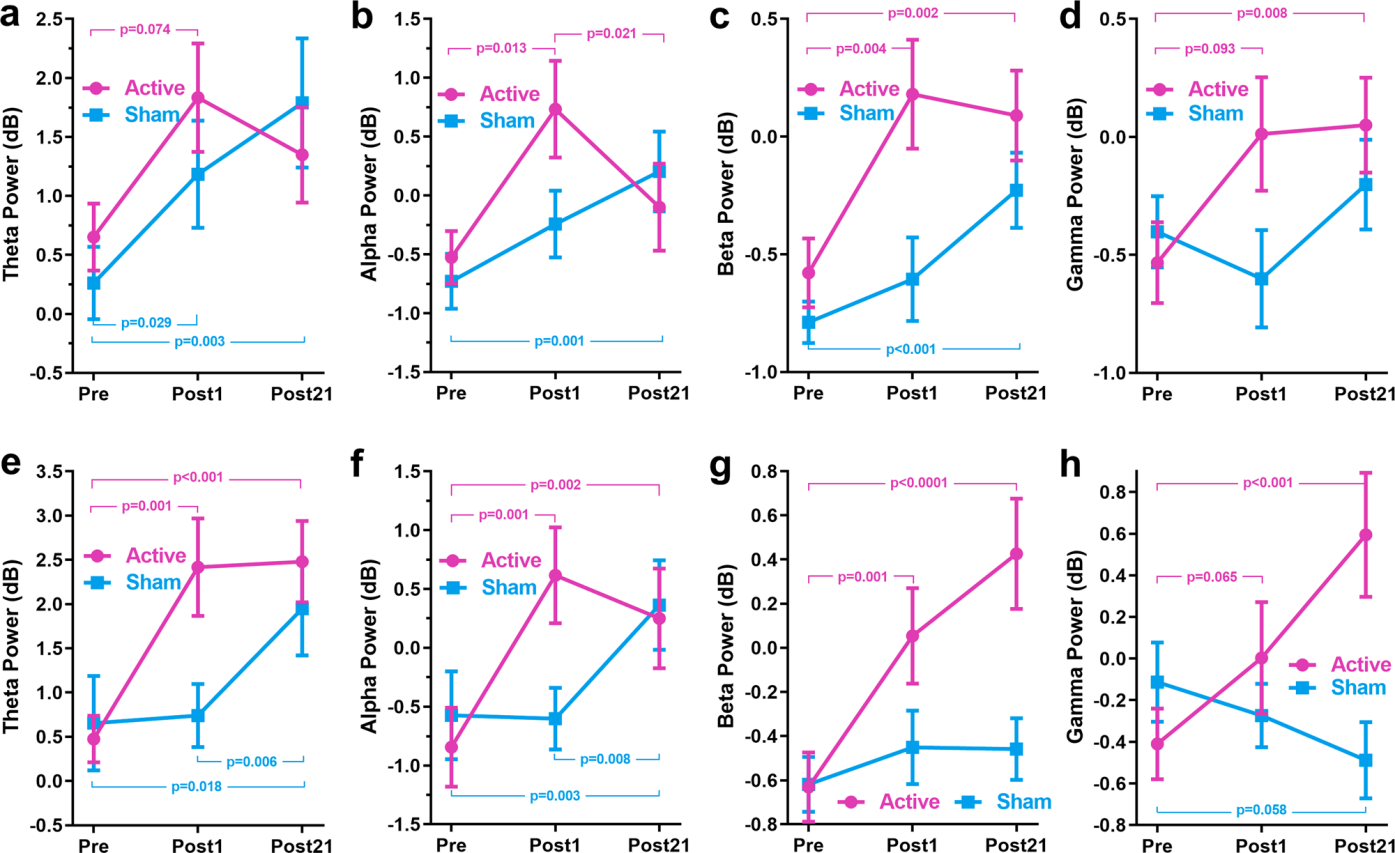
The frontal task-related EEG power measures in verbal 4- and 6-back tasks. **a**–**d** Task-related EEG power measures of theta, alpha, beta, and gamma bands for the 4-back task. **e**–**h** Task-related EEG power measures of theta, alpha, beta, and gamma bands for the 6-back task. *P*-values were determined by Wilcoxon signed-rank tests. Error bars depict SEM.

Figure 3 shows the grand-average frontal ERSP time-frequency distributions for active and sham groups at Pre- and Post-tests in both tasks. Theta power increases (ERS) and alpha, beta, and gamma power decreases (ERD) time-locked to stimulus onset appeared in the time window of interest in both groups at the Pre-test. Apparent changes in the preselected time-frequency windows of ERSP maps from Pre-test to Post-tests in both groups can be observed. More importantly, the changes showed apparent differences between groups. Quantitative statistics of the frontal power measures of the four bands are shown in Fig. 4, and the corresponding results of statistical analyses examining the effects of group and time are as follows.

#### 4-back

As shown in Table 1, ANCOVAs performed on the frontal power measures at post-tests in the 4-back task revealed significant effects of the Pre-test baseline in all the four bands, significant effects of group only in the gamma band, and insignificant effects of time in all the four bands after adjusting for baseline. The group-by-time interaction effect was significant only in the alpha band. Post hoc comparisons for the alpha band found a significant decrease from Post1- to Post21-test for the active group [t(16) = −3.045, ${p}_{{\rm{holm}}}$ = 0.029, Cohen’s d = −0.714], but no significant change was found for sham group [t(16) = 1.685, ${p}_{{\rm{holm}}}$ = 0.411, Cohen’s d = 0.395]. Mann-Whitney tests conducted on power measures at Pre-test confirmed no significant difference between groups in all the four bands [theta: M-W = 153.500, *p* = 0.361, Rank-Biserial Correlation = 0.195; alpha: M-W = 149.000, *p* = 0.445, Rank-Biserial Correlation = 0.164; beta: M-W = 161.000, *p* = 0.224, Rank-Biserial Correlation = 0.258; gamma: M-W = 118.000, *p* = 0.724, Rank-Biserial Correlation = −0.078]. Comparisons between Pre-test and Post1-test using Wilcoxon signed-rank tests revealed that the active group’s power measures significantly increased in beta band but not in theta, alpha, and gamma bands. In contrast, the sham group’s power measures in all four bands did not significantly change from Pre-test to Post1-test (see Table 2 and Fig. 4 panels a–d). Comparisons between Pre-test and Post21-test found significant increments in beta and gamma bands for the active group and in theta, alpha, and beta bands for the sham group (see Table 2 and Fig. 4 panels a–d).

#### 6-back

After adjusting for baseline using ANCOVAs performed on the frontal power measures in 6-back task, significant effects of Pre-test baseline and group were observed in all the four bands, but significant effects of time were found only in the alpha band, significant effects of group-by-time interaction were found in alpha and gamma bands (Table 1). Simple main effects analysis for alpha power measures showed that the active group had a significantly higher alpha power than the sham group at the Post1-test [*F*(1, 31) = 10.364, *p* = 0.003, ${\eta }_{p}^{2}$ = 0.263] but group effect was insignificant at the Post21-test [*F*(1, 31) = 0.087, *p* = 0.770, ${\eta }_{p}^{2}$ = 0.003]. Simple main effects analysis for gamma power measures showed that group effect was insignificant at the Post1-test [*F*(1, 31) = 1.796, *p* = 0.191, ${\eta }_{p}^{2}$ = 0.058], however, the active group had a significantly higher gamma power than the sham group at the Post21-test [*F*(1, 31) = 24.923, *p* < 0.0001, ${\eta }_{p}^{2}$ = 0.462]. Mann–Whitney tests conducted on the power measures at Pre-test confirmed no significant difference between groups for all the four bands [theta: M-W = 134.000, *p* = 0.838, Rank-Biserial Correlation =0.047; alpha: M-W = 115.000, *p* = 0.642, Rank-Biserial Correlation = −0.102; beta: M-W = 123.000, *p* = 0.867, Rank-Biserial Correlation = −0.039; gamma: M-W = 101.000, *p* = 0.323, Rank-Biserial Correlation = −0.211]. From Pre- to Post1-test, power measures of theta, alpha, and beta bands, but not the gamma band, significantly increased in the active group, however, power measures of all four bands did not significantly change in the sham group (see Table 2 and Fig. 4 panels e–h). The active group’s power measures also significantly increased from Pre-test to Post21-test in all four bands; in contrast, the sham group showed significant increments in alpha band but no significant change in theta, beta, and gamma bands (see Table 2 and Fig. 4 panel e–h).

In Summary, these statistical results indicate that the power measures showed an increasing trend from the Pre-test to the Post-tests, resulting in stronger theta ERS (increased theta oscillations) and weaker alpha, beta, and gamma ERDs (increased task-related alpha, beta, and gamma power measures) at Post21-test. But the important difference between groups is that the power measures of the active group increased faster/stronger than the sham group, which is manifested in the group differences at the Post1-test (e.g., theta, alpha, and beta in the 6-back task) and the significant increments from Pre- to Post1-test only found in the active group (e.g., alpha and beta in 4-back task and theta, alpha and beta in the 6-back task). These statistical findings incorporating the results shown in Fig. 4 suggest HD-tDCS over the left dlPFC facilitated and enhanced the training-induced rise of the active group’s task-related EEG activity from the Pre- to Post21-test.

### P300 GFP

**Fig. 5:**
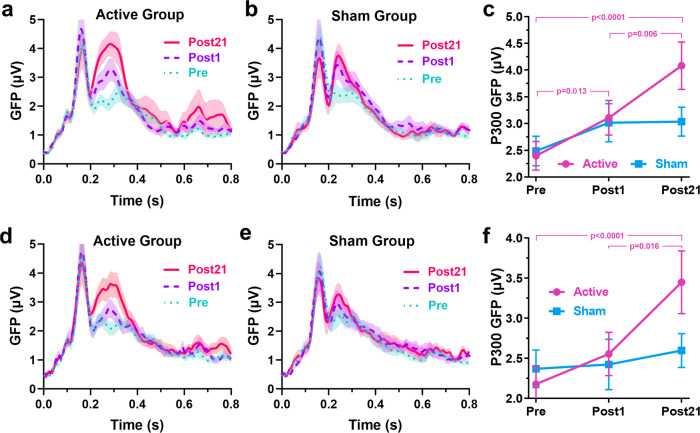
Statistics of P300 GFP for verbal 4- and 6-back tasks. **a**, **b** GFP curves in the verbal 4-back task of active and sham groups, respectively. **d**, **e** GFP curves in the verbal 6-back task of active and sham groups, respectively. **c**, **f** The average P300 GFPs of both groups in verbal 4- and 6-back tasks, respectively. Error bars depict SEM. *P*-values were determined by Wilcoxon signed-rank tests.

Greater changes of P300 GFP amplitudes (in the preselected time window) in the active group than in the sham group from the Pre- to Post21-test can be seen from the GFP curves in both tasks (Fig. 5a, b, d, e). Figure 5c, f shows the average P300 GFPs of both groups in 4-back and 6-back, respectively. The effects of the Pre-test baseline on the Post-tests P300 GFP were significant in both tasks, as revealed by mixed ANCOVAs. After adjusting for the baseline P300 GFPs, insignificant group effects in both tasks, significant time effect in 6-back, and significant group by time interaction effect in 4-back were observed (Table 1). Simple main effects analyses for P300 GFP of 4-back task showed that the group effect was not significant at Post1-test [*F*(1, 31) = 0.165, *p* = 0.687, ${\eta }_{p}^{2}$ = 0.006] but this effect was significant at Post21-test [*F*(1, 31) = 5.677, *p* = 0.024, ${\eta }_{p}^{2}$ = 0.164]. Mann–Whitney tests conducted on P300 GFP at Pre-test confirmed no significant difference between groups in both tasks [4-back: M-W = 123.000, *p* = 0.867, Rank-Biserial Correlation = −0.039; 6-back: M-W = 110.000, *p* = 0.515, Rank-Biserial Correlation = −0.141]. Comparisons between Pre-test and Post-tests showed significant increments from Pre- to Post1-test in the 4-back task and from Pre- to Post21-test in both tasks only for the active group’s P300 GFP (see Table 2 and Fig. 5 panel c and f). These findings support that active tDCS induced lasting after-effects and enhanced the P300 response in both tasks, as demonstrated by the group differences at Post-tests and the significant increases in the active group’s P300 GFP from Post1- to Post21.

### Relation between baseline and changes after training

Next, we examined how training-related changes in behavioral and EEG measures were related to individual differences in the corresponding measures before training. Correlation analyses for WM performance measures revealed that individual differences in both groups in the magnitude of improvements in *d*-primes and reaction times at post-tests were negatively correlated with pre-training *d*-primes and RTs in both tasks: those with lower pre-training performance levels showed greater performance gains. Except for the sham group’s improvements in *d*-prime in the 4-back task, the correlations between improvements and baselines were significant in all other conditions (*p*s < 0.05; see Supplementary Fig. 1 and Supplementary Table 5). Significant group differences were found only in the correlation between improvements and baselines in *d*-prime in the 4-back task at the Post1-test.

**Fig. 6:**
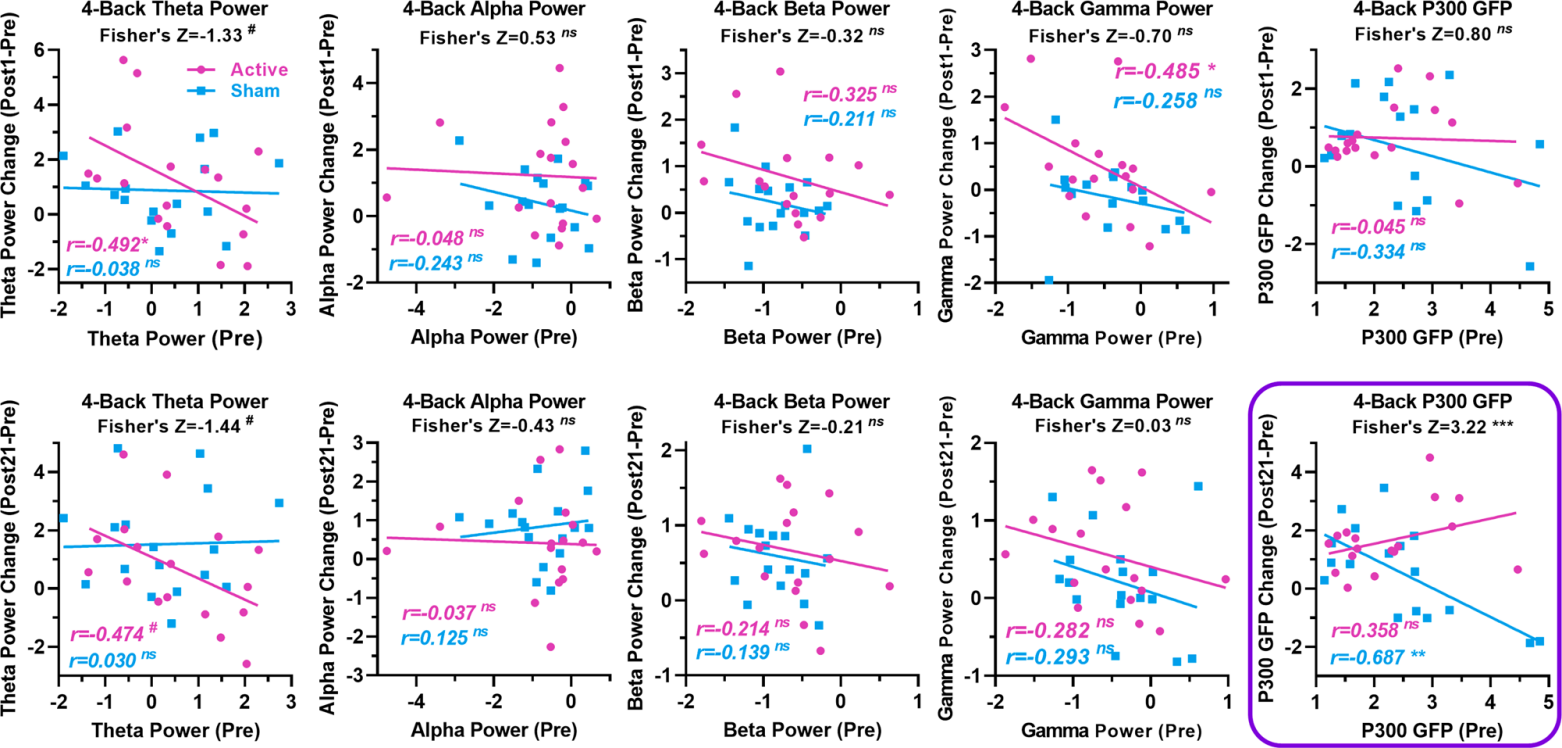
EEG dynamics prior to training correlate with changes in EEG dynamics in the verbal 4-back task. The panel with a purple box highlights the between-group difference in correlation coefficients. *P*-values were determined by Fisher’s Z-tests. (^#^*p* < 0.1; *p < 0.05; **p < 0.01; ***p < 0.01; ns not significant).

**Fig. 7:**
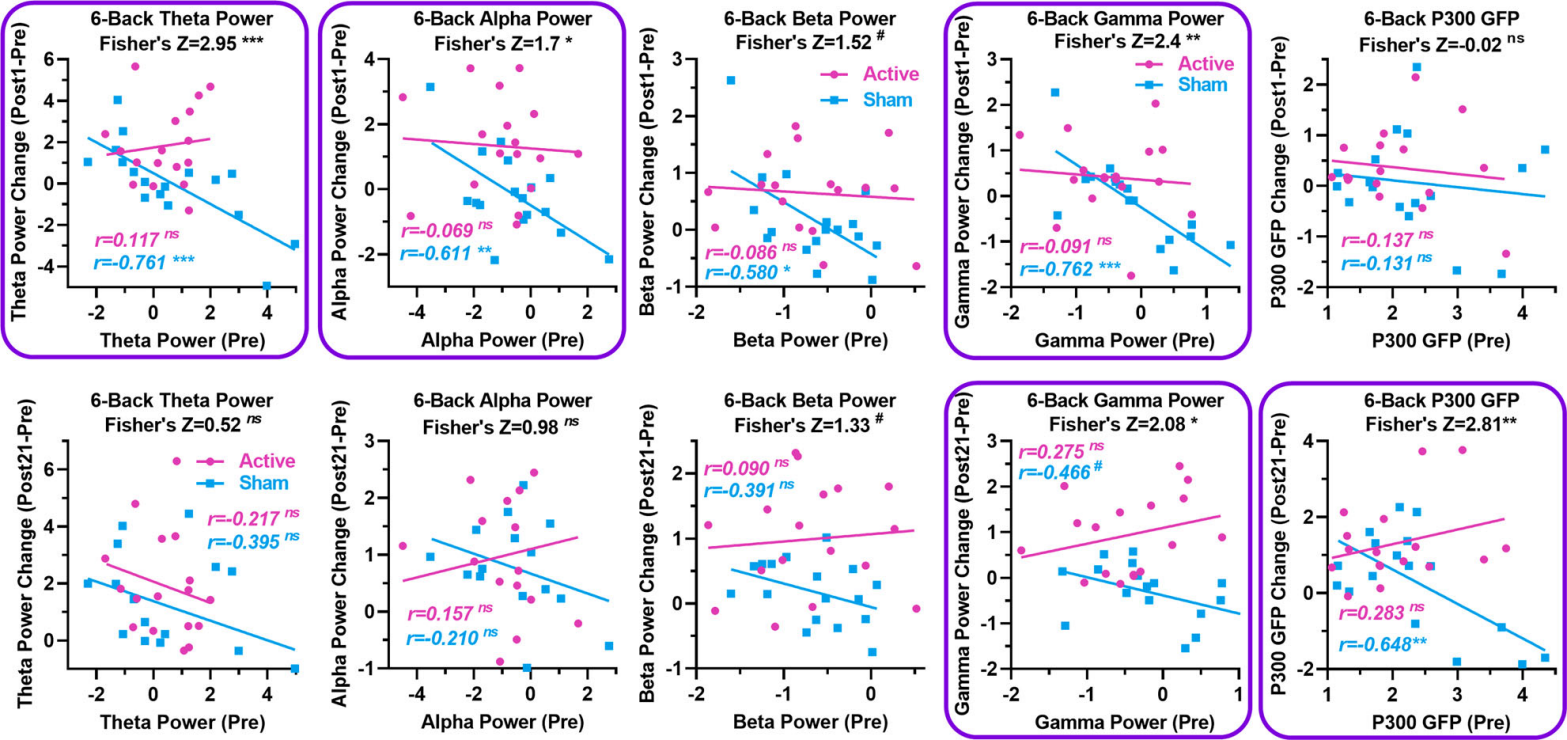
EEG dynamics prior to training correlate with changes in EEG dynamics in the verbal 6-back task. The panels with a purple box highlight between-group differences in correlation coefficients. *P*-values were determined by Fisher’s Z-tests. (^#^*p* < 0.1; *p < 0.05; **p < 0.01; ***p < 0.001; ns not significant).

Figures 6 and 7 show the results of the correlation analysis between pre-training EEG dynamics and post-training changes in EEG dynamics. In the 4-back task, changes in the active group’s theta and gamma power measures at Post1-test and the sham group’s P300 GFP at Post21-test showed significant negative associations with their baselines, respectively, but a significant group difference in the correlation coefficient was found only for the P300 GFP at Post21-test. The associations between baselines and changes in P300 GFP in the active group were positive but not significant at Post21-test. In contrast, these associations were negative and strong in the sham group (see Fig. 6 and Supplementary Table 5). In the 6-back task, there were strong negative associations between baseline and changes in theta, alpha, and gamma power measures at Post1-test and in the P300 GFP at Post21-test for the sham group. More importantly, significant group differences have been found in the associations between baselines and changes from Pre- to Post1-test in theta, alpha, and gamma power and in the associations between baselines and changes from Pre- to Post21-test in gamma power and P300 GFP (see Fig. 7 and Supplementary Table 5).

### Learning curves

The learning curves and the corresponding results of learning rates are shown in Supplementary Fig. 2. With respect to the performance variations during training, both groups showed improvements in their WM capacity as quantified by the capacity index (*K*) over the training course, whereas only active group exhibited significant training effect according to the results of two-way mixed ANOVA. From the aspect of learning rate, our results also indicate significant learning effects with a large effect size across the 10-day training for active group but not for sham group. One particularly notable finding about learning curves in the present study is that the active group only showed significant training effects in the earlier phase and reached a capacity plateau after training for 6 days, possibly due to a ceiling effect, while the sham group’s capacity index exhibited a much slower rising across the training course.

### Safety, tolerability, and blinding

No participant reported severe adverse effects like headache, sleepiness, trouble concentrating, and psychological symptoms during/after any tDCS sessions or withdrew from the study due to significant discomfort. The main adverse effects reported in this study were tingling, itching, and burning sensation during the stimulation period, but none of the reported intensity ratings exceeded 7 for any participant, suggesting that the tDCS intervention was well-tolerated. At the end of the prolonged training session, the proportions of subjects correctly guessing the tDCS condition were 41% and 35% for the active and sham groups, respectively [chi-square test: χ^2^(1) = 0.583, *p* = 0.445], suggesting adequate blinding according to previous studies^43,44^.

## Discussion

This study investigated whether and how multi-session anodal tDCS over the left dlPFC, applied during WMT, can induce or enhance sustained changes in WM-related neural circuitry after the end of training, using task-related EEG dynamics. Our analysis yielded four main findings. First, our results confirmed previous findings that tDCS over the left dlPFC can enhance WMT and produce lasting behavioral benefits. Second, tDCS accelerated and enhanced changes in WMT-induced ERD/ERS variations. Third, and more importantly, tDCS induced persistent neurophysiological aftereffects as manifested by the enhanced task-related EEG power and P300 responses three weeks after training. Forth, tDCS may have enhanced the training effect mainly at the earlier training phase according to the results of learning curves. Our findings demonstrate that tDCS paired with WMT can facilitate and enhance training-induced neurophysiological effects, produce lasting after-effects during the post-training skill consolidation phase, and provide new insights into the neurobiological mechanisms underlying the behavioral effects of multi-session tDCS.

Our results on behavioral measures provide evidence that WMT leads to significant and lasting improvements in WM performance in both groups. Importantly, anodal tDCS over the left dlPFC enhanced the behavioral benefits of WMT, but this effect was found only in WM accuracy, as manifested by the superior *d*-prime of the active group at post-tests. We also found significant and strong negative associations between baseline and training gains in *d*-primes and RTs in most cases. This finding suggests a greater benefit of WMT for subjects with lower WM performance and is consistent with previous studies^45^. The absence of a modulation effect of tDCS on the association between baseline and training gains in behavioral measures could be attributed to the fact that high-intensity training greatly improved behavioral performance in both groups.

The differences in changes in task-related EEG power measures from Pre- to Post-tests between the two groups suggest that active tDCS enhanced or facilitated WMT-induced increments in frontal task-related theta, alpha, beta, and gamma oscillations during update and readout in WM processing. Specifically, compared to baseline, these oscillations were significantly enhanced in the active group at the Post1-test, but significant changes in these oscillations of the sham group were only found at the Post21-test. Frontal theta has been considered a signature of successful WM manipulation and plays an important general integrative role in the organization of cognitive processes^21,46^. The increases in task-related theta oscillations after training may indicate an improved ability to integrate different cognitive resources. ERD/ERS in alpha and beta bands are related to the decrease/increase in firing synchrony of neurons involved in frequency-specific event-related brain processes, respectively, with ERD characterizing cortical areas involved in task-relevant processing and ERS marking cortical areas in an idle state^47^. Within the theoretical framework of neural efficiency, increasing task-related alpha and beta oscillations reflect the reduced amount of neural resources that need to be engaged during problem-solving, thus increasing neural efficiency in information processing^24,48,49^. With regard to higher frequency bands, increased task-related frontal gamma oscillatory activity has been relevant to increased top-down attentional processes and would lead to better performance while performing WM tasks especially during higher WM loads^50–52^. Significant increases of gamma oscillatory activity from Pre- to Post21-test were only found in the active group may suggest lasting enhancement effects of multi-session tDCS in top-down attentional ability. Taken together with the group differences in these oscillatory activities, our findings may suggest that WMT-induced enhancements in neural efficiency within WM-associated neural substrates may require a longer period of skill consolidation after the training ends; however, anodal tDCS may have facilitated this process and enhanced top-down attentional control ability in the active group, resulting in lasting behavioral performance enhancements.

In addition to the findings in task-related oscillations, we found that P300 GFPs increased significantly after training with large effect sizes in the active group but not in the sham group. The interesting finding is that the significant increases in P300 GFP in the active group occurred from Post1 to Post21, suggesting long-lasting aftereffects of tDCS on neurocircuits supporting WM processing during the skill consolidation phase. The P300 reflects attentional allocation and memory updating processes during sensory input processing^53^, and the increasing GFP reflects increased synchronous neuronal activation and population size^54^. Previous studies have found that the n-back task may involve some dual-tasking, and the P300 response decreases with increasing WM load in n-back tasks^26^. The increasing P300 GFP can be interpreted in terms of a stronger engagement of neural resources in sensory input processing during the n-back task and may reflect less effortful WM processing due to increased neural efficiency of WM-related neural substrates, thus indicating improved efficiency of attentional allocation and more neural resources available to complete the task^55,56^.

**Fig. 8:**
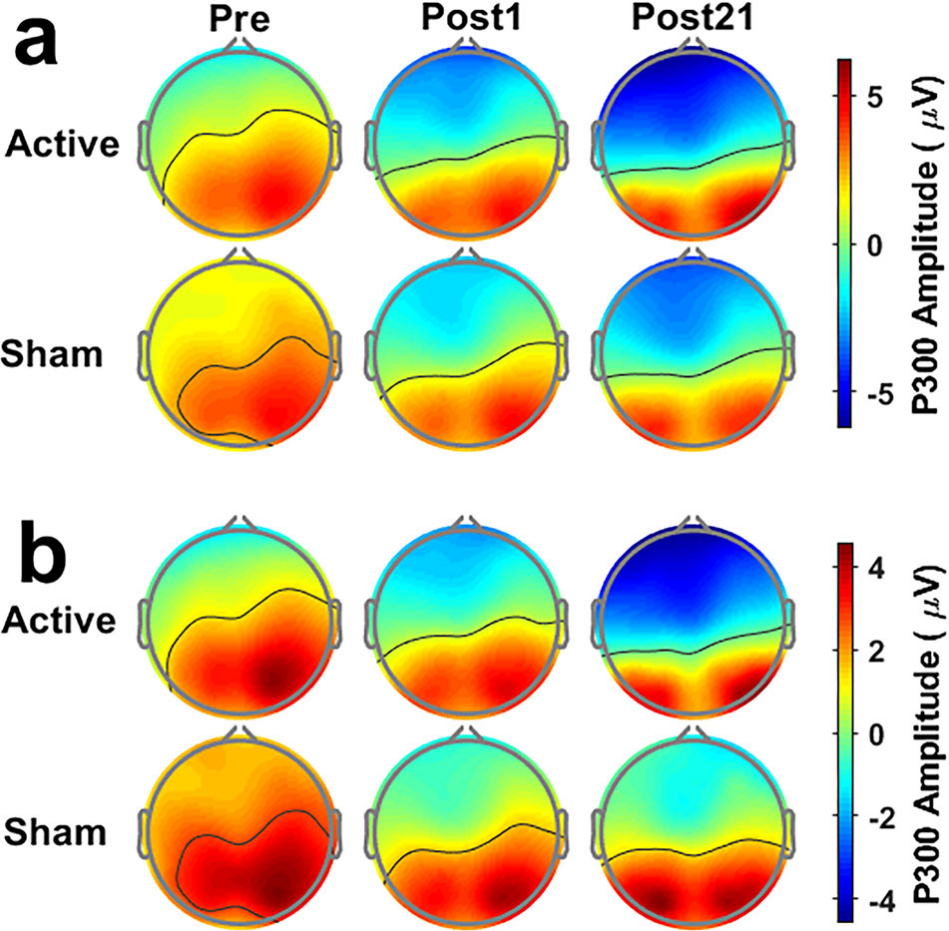
Topological plots of the P300 component. **a** verbal 4-back task. **b** verbal 6-back task.

Topological plots of the P300 component (Fig. 8) support that the changes in P300 responses are not simply changes in the amplitude of specific channels or local brain regions, but changes in the global distribution. This proves the rationality of analyzing the P300 response using GFP rather than the amplitude of local channels. The changes in P300 GFP from Post1 to Post21 in the active group are primarily attributed to the increased anterior negativity as seen in the topological plots. A more negative P300 deflection at anterior scalp regions has been associated with higher WM capacity^57^ and lower WM load^26^. The topological characteristics of P300 showed a widespread positivity at Pre-test, but an anterior negativity was shown along with a posterior positivity at Post-tests, which implies changes in the primary neuropsychological origins of the observed P300 components. These alterations may suggest a potential explanation for heightened sensory responses due to reduced distraction or resource competition from WM operations, consistent with increased neural efficiency as manifested by the changes in ERD/ERS, P300 GFP, and WM performance. Conforming to the findings of P300 GFP, only the active group showed more notable changes at Post21 but not Post1, suggesting lasting effects of multi-session tDCS on WM-related neurocircuits.

Correlation analysis between baseline and changes in task-related EEG dynamics revealed group differences. Specifically, where group differences were found, changes in the sham group were strongly negatively associated with baseline, while associations in the active group were not significant. The strong negative associations observed in the sham group should be a manifestation of “less room for change” in subjects with stronger task-related theta, alpha, and gamma oscillations, as well as P300 responses, and point to greater changes in subjects with weaker task-related EEG responses, which could be associated with lower neural efficiency according to the aforementioned mechanisms of task-related EEG dynamics and neural efficiency. In particular, the significant group differences and the nonsignificant associations in the active group suggest that tDCS over the left dlPFC broke the “less room for change” effect in subjects with stronger task-related EEG responses. This effect of tDCS is particularly evident in the changes in P300 GFP at the Post21 test, further confirming the sustained effects of tDCS.

Taken together, the most encouraging findings from the behavioral and neurophysiological measures are the facilitatory effects and the lasting aftereffects of tDCS on task-related EEG responses. A possible interpretation of these effects may be that tDCS can facilitate the automatization of cognition, reorganization, and consolidation effects of neuroplasticity over time^58,59^. Previous studies have suggested that a sufficient explanation for such long-term or chronic effects of tDCS should be metaplasticity, rather than the long-term potentiation and long-term depression phenomena used to explain the first direct consequence of tDCS^60^. Metaplasticity has been defined as a higher-order form of plasticity that can enable or inhibit plasticity induction, stabilize synapses, and homeostatically regulate cellular activity^60,61^. When brain stimulation is paired with repeated cognitive training that can induce synaptic plasticity, metaplasticity modulates synaptic plasticity and extends the time window for associative plasticity, thereby increasing brain stimulation-induced aftereffects on cortical excitability or learning^60,62^. In the present study, anodal tDCS over the left dlPFC may have modulated synaptic plasticity of WM-related neural substrates by enhancing the effects of WMT and producing long-lasting neurobehavioral improvements in WM tasks. Given the observed effects and the theoretical underpinnings of the neurophysiological measures, the results of this study are particularly noteworthy for investigating the effects of tDCS on cognitive training.

In conclusion, anodal tDCS over the left dlPFC facilitated WMT-induced lasting neurocircuit reorganization and consolidation effects. The behavioral benefits may persist and the neural changes may even be enhanced by anodal tDCS during consolidation and after weeks of no exposure to tDCS or WM tasks. These findings provide evidence for the lasting neurophysiological effects of tDCS during WMT and provide essential insights into the neurocognitive mechanisms underlying multi-session tDCS. The future challenge is to determine how long the neurophysiological aftereffects might last and whether these effects can be reproduced in healthy and clinical populations of different ages. Future investigation of the transferability of tDCS-related improvements to untrained tasks could also yield valuable insights and provide valuable guidance for the application of tDCS in cognitive training.

## Methods

### Participants

Thirty-four graduate/undergraduate students (22.50 ± 1.52 years old, including 18 males) volunteered to participate in this study with payments. All participants had normal or corrected-to-normal vision and no self-reported history of mental or neurological disorders or substance abuse. All participants were screened for tDCS contraindications to ensure that they did not have metal implants or sensitive skin. We used a between-subjects design and randomly assigned participants to active or sham HD-tDCS groups with no significant difference in gender [chi-squared test: χ^2^(1) = 1.889, *p* = 0.169] and age [active: 22.82, 95% CI = [22.14–23.51]; sham: 22.18, 95% CI = [21.32–23.03]; t(32) = 1.249, *p* = 0.221]. Participants had no prior experience with tDCS and were blinded to their group. The study was approved by the Ethics Committee of Tianjin University and conducted in accordance with the Declaration of Helsinki. Written informed consents were obtained from all participants.

### Experimental procedure

The experiment in this study consisted of ten consecutive training sessions and three test sessions, as shown in Fig. 1a. During the training phase, all participants received ten consecutive days of verbal WM training along with either active or sham tDCS. On each day of training, participants were instructed to perform one block of visual verbal 4- and 6-back tasks in addition to four blocks of load-adaptive visual verbal *N*-back tasks within 30 min. Load-adaptive verbal *N*-back means that the current session’s load factor *N* is adjusted according to the previous session’s performance. The load factor in the current session would be *N* + 1 if participants achieved response accuracies above 85% in verbal *N*-back in the last session. Otherwise, the load factor would remain the same as in the previous session. No maximum load factor was set in this study.

Without applying tDCS, WM performance was assessed along with EEG recording at least one day before the first training session (Pre-test session) and one day (Post1-test session) and three weeks (Post21-test session) after the end of the training. In each testing session, participants were instructed to perform two blocks of visual verbal 4- and 6-back tasks in random order. EEG data were recorded along with the tasks in the Pre-, Post1-, and Post21-tests. Mean reaction time and the psychometric *d*-prime (*d’* = z(hit rate)−z(false alarm rate))^63^ were employed as the performance measures for the Pre-, Post1-, and Post21-tests.

### WM tasks

In this study, participants’ WM capacity was trained using an adaptive visual verbal *N*-back task and tested using visual verbal 4- and 6-back tasks. The *N*-back task is a classic test of WM in which participants are required to recall items from further back in time and to compare the current item with the one that was presented *N* steps earlier. The difficulty of *N*-back tasks can be easily adjusted by increasing or decreasing the load factor *N*. In the present study, the items of the verbal *N*-back task were the ten uppercase consonant letters. All tasks were presented in the center of a monitor and were implemented in PsychoPy^64^. Each block contained 80 trials, including 40 match trials and 40 non-match trials. In each trial, a letter was presented for 500 ms, followed by a cross “+“ for 3000 ms. During the trial duration (3.5 s), participants were instructed to press the left arrow key if the current item matched the one presented *N* steps earlier, or the right arrow key if it did not match.

### HD-tDCS protocol

In this study, sham or active tDCS was applied using the Starstim system (Neuroelectrics, Spain) via circular saline-soaked sponge electrodes (3.5 cm in diameter) organized in a 4 × 1 HD montage. As shown in Fig. 1b, the stimulation electrodes were embedded in a cap with the international 10–20 EEG system and configured to achieve maximum focality for the left dlPFC based on a computational model of the cortical electrical field estimated using the SimNIBS software^65^. The anodal electrode was placed over F3 and surrounded by four return electrodes placed over Fp1, Fz, C3, and F7 (the 10–20 international EEG system) according to the neurophysiological study by Hill et al.^42^. In this study, an online tDCS strategy was used, i.e., tDCS was started simultaneously with WMT for each session. The active group received 2 mA tDCS (current density = 0.104 mA/cm^2^) for 25 min (30 s ramp-up, 30 s ramp-down). The sham group received only a 30-s ramp-up and 30-s ramp-down to induce physical sensation without stimulating the cortical areas below the anode during training. At the end of each training session, subjects rated the intensity of side effects (tingling, itching, and burning) of HD-tDCS using a 10-point Likert scale. To assess blinding integrity, subjects were asked to guess their assigned tDCS condition at the end of the last training session.

### EEG recording and processing

60-channel EEG data were recorded using the Neuroscan SynAmps 2 system with a sampling rate of 1000 Hz at Pre, Post1, and Post21 visits. EEG was referenced to the left mastoid during recording and re-referenced to the average of the left and right mastoids for offline analysis. EEG data were then band-pass filtered from 0.5 to 45 Hz and downsampled to 500 Hz. EEG epochs with obvious noise were manually removed. The average numbers of EEG epochs included in the following analysis were around 130 with no significant difference across groups and testing sessions for all the tasks (see Supplementary Fig. 3). Bad channels were visually identified and replaced with spherical spline interpolation. Blink and eye movement artifacts were corrected using Independent Component Analysis (ICA) in EEGLAB^66^. EEG epochs including 500 ms before stimulus to 2000 ms after stimulus were extracted from the denoised continuous EEG for ERP and event-related spectral perturbation (ERSP) analyses.

ERSPs (in dB) were estimated for task-related EEG in [2 Hz, 45 Hz] with baseline correction using the EEGlab *newtimef()* function with the Morlet wavelet. We focused on task-related EEG power measures in the frontal region by averaging ERSP across all frontal channels (Fp1, Fpz, Fp2, AF3, AF4, F7, F5, F3, F1, Fz, F2, F4, F6, and F8) to avoid spatial bias of single-channel analysis, as *N*-back has been found to broadly activate the frontal lobe, especially dlPFC, anterior PFC, and vlPFC^67^. The task-related EEG power of the theta (4–8 Hz), alpha (8–14 Hz), beta (15–30 Hz), and gamma (31–45 Hz) bands were extracted by averaging across time points and frequencies for the four bands of interest for statistical analyses. To ensure an unbiased selection of the time window of interest for power analysis, the time window between 300 and 1000 ms was used in the following task-related EEG power analysis. We chose this interval to account for the time range for update and readout in WM processing and to exclude the contribution of visual processing in visual *N*-back tasks^68^.

For P300 analysis, average ERP waveforms were obtained by averaging across trials for each condition and participant after baseline correction by subtracting the mean amplitudes in the −200 to 0 ms pre-stimulus interval. Given that distributed sources generate the P300 ERP, we sought to obtain a global, reference-free, and (with respect to the underlying sources) hypothesis-free measure of P300 event-related activity. Global field power (GFP), which can measure global electric field strength modulations^69^ was calculated for the ERP waveforms by calculating the spatial root mean square across all electrodes, resulting in a reference-independent metric reflecting the global ERP strength in the scalp domain. GFP globally reflects the number of neuronal elements activated synchronously^54^, assuming that high standard deviation among channels corresponds to increased activity. P300 GFP was the more stable P300 measure compared to amplitudes obtained from individual electrode positions (e.g., Pz). Therefore, P300 GFP was selected as the main P300 ERP variable of interest in subsequent statistical analyses. To determine the time window of the P300, we calculated the mean GFP across groups and trials and selected the 90 ms time window around the peak in 250–400 ms. This procedure resulted in a time window of interest for P300 GFP of 240–330 ms.

### Statistical analyses

The mean P300 GFP measure and the mean task-related EEG power measures of theta, alpha, beta, and gamma bands were obtained by averaging over the preselected time windows of interest and taken over to baseline-adjusted analysis of covariance (ANCOVA) models, respectively. ANCOVA was employed to compare groups post-intervention since ANCOVA models are more powerful as they can account for baseline imbalance and correlation between baseline and post-intervention measures, increase statistical power and minimize biases^70^. To address our research question of whether participants’ WMT-induced outcomes on WM performance measures (the *d*-prime and RT) change in response to the tDCS intervention, we conducted mixed ANCOVAs on *d*-prime and RT of the post-tests with group (active, sham) as a between-subject factor, time (Post1- and Post21-tests) as a within-subject factor, and Pre-test WM performance as a covariate of no interest to control for pre-existing differences. To address whether the WM-related EEG dynamics, the task-related EEG power measures and the P300 GFP, changed in response to tDCS intervention, statistical analyses were performed on the frontal EEG power measures and the P300 GFP of the post-tests (Post1- and Post21-tests) by conducting mixed ANCOVAs with the Pre-test EEG power measures or P300 GFP as a covariate to control for pre-existing differences in WM-related EEG dynamics. To verify baseline differences between groups, non-parametric Mann–Whitney (M-W) tests at the Pre-test determined whether the two groups (active vs. sham) demonstrated similar or different baseline WM performance and EEG dynamics levels. Comparisons using Wilcoxon signed-rank (W-SR) tests between the Pre-test and the Post1- and Post21-tests for the active (or sham) group examined whether and how 10 days of training paired with active (or sham) tDCS lead to changes in WM performance and EEG dynamics. The significance level was set to 0.05 for the ANCOVAs and 0.0125 (=0.05/4, adjusted for multiple comparisons) for the Wilcoxon signed-rank tests between Pre-test and Post-tests. The Holm-Bonferroni correction was applied in post hoc analyses if applicable.

To further address whether pre-training levels of WM performance and task-related EEG dynamics influence the magnitude of their changes with WMT, we examined how changes were associated with individual differences in subjects’ baseline levels of neurobehavioral measures using Pearson’s correlation. Fisher’s Z-test using the VassarStats website (http://vassarstats.net/rdiff.html) was used to test the difference in correlation coefficients between the active and sham groups to examine whether tDCS moderated the relationship between neurobehavioral measures at pre-test and the corresponding changes after training.

To examine the training process, the WM capacity index (*K*) defined as the multiplication of the load factor *N* with the corresponding *d*-prime (*K* = N*d’) was employed to measure participants’ WM capacity during training. Two-way mixed ANOVA was performed on *K* value with group (active group vs. sham group) as the between-participant factor, and training session (session 1, session 2, …, session 10) as the repeated measures. The Greenhouse-Geisser correction was employed if the assumption of sphericity was violated (Mauchly’s test, *p* < 0.05). The learning rate of each subject was determined by the slope of linear regression over session-wise *K* value: (i) across all 10 days (overall learning rate), (ii) across earlier training phase from day 1 to day 5 (earlier learning rate), (iii) across later training phase from day 6 to day 10 (later learning rate). Two-way (group × training phase) mixed ANOVA was employed to compare learning rates between groups and between training phases.

Before applying the ANCOVAs and the ANOVAs, the distributions were checked for normality (Shapiro–Wilk test) and if any were non-normal (*p* < 0.05), the Box-Cox transformation was used to transform them into a normal distribution. All the analyses, including estimates of effect sizes (Cohen’s d for *t*-test; Ranked-Biserial Correlation for Mann–Whitney test and Wilcoxon signed-rank test; partial *η*^2^, ${\eta }_{p}^{2}$, for ANCOVA and ANOVA), were conducted in JASP (version 0.16.2).

### Supplementary information

supplement materials

reporting-summary

## Data Availability

Anonymized data supporting the results of this study are available upon request from the corresponding authors for non-commercial use without restriction, as one of our funders (Open Funding Project of National Key Laboratory of Human Factors Engineering) does not allow commercial use of the data.

## Code availability

The codes used to analyze the data were based on the functions of the public toolbox EEGLAB and are available from the corresponding authors upon request for academic purposes.
