# Supplementary material for: Lasting enhancements in neural efficiency by multi-session transcranial direct current stimulation during working memory training
Source: NPJ Sci Learn. 2023 Nov 2;8:48. doi: 10.1038/s41539-023-00200-y (PMC10622507; doi:10.1038/s41539-023-00200-y)
Supplement: Supplementary file 1 — supplement materials [file 41539_2023_200_MOESM1_ESM.pdf]

Supplementary materials for "Lasting enhancements in neural efficiency by multi-session transcranial direct current stimulation during working memory training"

Yufeng Ke[1,2,*], Shuang Liu[1,2,*], Long Chen[1,2], Xiashuang Wang[3] and Dong Ming[1,2,*]

[1] Academy of Medical Engineering and Translational Medicine, Tianjin International Joint Research Centre for Neural Engineering, and Tianjin Key Laboratory of Brain Science and Neural Engineering, Tianjin University, Tianjin, PR China.

[2] Haihe Laboratory of Brain-computer Interaction and Human-machine Integration, Tianjin, PR China.

[3] The Second Academy of China Aerospace Science and Industry Corporation, Beijing, PR China.

[*] Author to whom any correspondence should be addressed.

E-mails: clarenceke@tju.edu.cn (Y.K.), richardming@tju.edu.cn (D.M.), and shuangliu@tju.edu.cn (S.L.)

Supplementary Table 1. Unadjusted (Unadj., mean and standard deviation) and baseline-adjusted (Adj., estimated marginal mean and standard error) descriptive statistics for *d*-primes and reaction times in verbal 4- and 6-back tasks at Post1- and Post21-tests.

Condition	*d*-prime				reaction time (s)			
	Verbal 4-back		Verbal 6-back		Verbal 4-back		Verbal 6-back	
	Unadj.	Adj.	Unadj.	Adj.	Unadj.	Adj.	Unadj.	Adj.
Post1								
Active	4.124 (0.390)	4.112 (0.148)	3.446 (0.502)	3.427 (0.139)	0.575 (0.168)	0.584 (0.038)	0.672 (0.157)	0.696 (0.038)
Sham	3.464 (0.781)	3.475 (0.148)	2.897 (0.624)	2.916 (0.139)	0.597 (0.197)	0.589 (0.038)	0.684 (0.209)	0.659 (0.038)
Post21								
Active	4.163 (0.336	4.147 (0.148)	3.143 (0.662)	3.111 (0.171)	0.595 (0.157)	0.603 (0.031)	0.713 (0.169)	0.731 (0.041)
Sham	3.506 (0.835	3.522 (0.148)	2.725 (0.742)	2.758 (0.171)	0.608 (0.154)	0.600 (0.031)	0.680 (0.197)	0.662 (0.041)

Supplementary Table 2. Unadjusted (mean and standard deviation) and baseline-adjusted (estimated marginal mean and standard error) descriptive statistics for task-related EEG power measures in verbal 4-back task at Post1- and Post21- tests.

Condition	Theta power		Alpha power		Beta power		Gamma power	
	Unadjusted	Adjusted	Unadjusted	Adjusted	Unadjusted	Adjusted	Unadjusted	Adjusted
Post1								
Active	1.833 (1.838)	1.733 (0.442)	0.733 (1.646)	0.666 (0.325)	0.181 (0.927)	0.132 (0.205)	0.012 (0.963)	0.037 (0.220)
Sham	1.185 (1.823)	1.285 (0.442)	-0.243 (1.135)	-0.176 (0.325)	-0.605 (0.711)	-0.556 (0.205)	-0.602 (0.826)	-0.626 (0.220)
Post21								
Active	1.350 (1.628)	1.230 (0.458)	-0.099 (1.483)	-0.199 (0.280)	0.089 (0.764)	0.008 (0.154)	0.050 (0.803)	0.095 (0.165)
Sham	1.789 (2.192)	1.908 (0.458)	0.203 (1.357)	0.302 (0.280)	-0.228 (0.641)	-0.146 (0.154)	-0.202 (0.762)	-0.247 (0.165)

Supplementary Table 3. Unadjusted (mean and standard deviation) and baseline-adjusted (estimated marginal mean and standard error) descriptive statistics for task-related EEG power measures in verbal 6-back task at Post1- and Post21- tests.

Condition	Theta power		Alpha power		Beta power		Gamma power	
	Unadjusted	Adjusted	Unadjusted	Adjusted	Unadjusted	Adjusted	Unadjusted	Adjusted
Post1								
Active	2.417 (2.203)	2.454 (0.437)	0.617 (1.627)	0.685 (0.297)	0.054 (0.866)	0.057 (0.184)	0.003 (1.076)	0.066 (0.211)
Sham	0.741 (1.425)	0.703 (0.437)	-0.602 (1.051)	-0.670 (0.297)	-0.451 (0.668)	-0.454 (0.184)	-0.274 (0.611)	-0.337 (0.211)
Post21								
Active	2.480 (1.839)	2.535 (0.429)	0.251 (1.694)	0.364 (0.274)	0.426 (0.999)	0.431 (0.168)	0.595 (1.191)	0.730 (0.190)
Sham	1.949 (2.117)	1.894 (0.429)	0.363 (1.521)	0.249 (0.274)	-0.459 (0.561)	-0.464 (0.168)	-0.498 (0.732)	-0.624 (0.190)

Supplementary Table 4. Unadjusted (mean and standard deviation) and baseline-adjusted (estimated marginal mean and standard error) descriptive statistics for P300 GFP of verbal 4- and 6-back tasks at Post1- and Post21- tests.

Condition	P300 GFP			
	Verbal 4-back		Verbal 6-back	
	Unadjusted	Adjusted	Unadjusted	Adjusted
Post1				
Active	3.108 (1.292)	3.141 (0.279)	2.553 (1.078)	2.632 (0.233)
Sham	3.014 (1.423)	2.981 (0.279)	2.423 (1.255)	2.344 (0.233)
Post21				
Active	4.082 (1.773)	4.111 (0.328)	3.448 (1.566)	3.512 (0.283)
Sham	3.036 (1.087)	3.007 (0.328)	2.597 (0.849)	2.533 (0.283)

Supplementary Table 5. Results of correlation analyses between baseline and changes after training in behavioral and EEG measures.

Behavioral/EEG Measure		Active Group		Sham Group		Active vs. Sham	
		r	*p*	*r*	*p*	Fisher' Z	*p*
Verbal 4-back, Post1-Pre	D-prime	**-0.908**	**<0.0001**	-0.447	0.0721	**-2.74**	**0.0031**
	RT	**-0.766**	**0.0003**	**-0.502**	**0.0402**	-1.21	0.1132
	theta power	**-0.492**	**0.0446**	-0.038	0.8837	-1.33	0.0918
	alpha power	-0.048	0.8534	-0.243	0.3466	0.53	0.2981
	beta power	-0.325	0.2039	-0.211	0.4164	-0.32	0.3745
	Gamma power	**-0.485**	**0.0486**	-0.258	0.3180	-0.70	0.2420
	P300 GFP	-0.045	0.8632	-0.334	0.1900	0.80	0.2119
Verbal 6-back, Post1-Pre	D-prime	**-0.821**	**<0.0001**	**-0.766**	**0.0003**	-0.39	0.3483
	RT	**-0.637**	**0.0060**	**-0.496**	**0.0428**	-0.55	0.2912
	theta power	0.117	0.6552	**-0.761**	**0.0004**	**2.95**	**0.0008**
	alpha power	-0.069	0.7926	**-0.611**	**0.0092**	**1.70**	**0.0446**
	beta power	-0.086	0.7424	**-0.580**	**0.0147**	1.52	0.0643
	Gamma power	-0.091	0.7273	**-0.762**	**0.0004**	**2.40**	**0.0082**
	P300 GFP	-0.137	0.6008	-0.131	0.6165	-0.02	0.4920
Verbal 4-back, Post21-Pre	D-prime	**-0.927**	**<0.0001**	-0.356	0.1604	**-3.34**	**0.0004**
	RT	**-0.793**	**<0.0001**	**-0.699**	**0.0018**	-0.56	0.2878
	theta power	-0.474	0.0547	0.030	0.9092	-1.44	0.0750
	alpha power	-0.037	0.8875	0.125	0.6331	-0.43	0.3336
	beta power	-0.214	0.4085	-0.139	0.5945	-0.21	0.4169
	Gamma power	-0.282	0.2731	-0.2926	0.2544	0.03	0.4880
	P300 GFP	0.358	0.1586	**-0.687**	**0.0023**	**3.22**	**0.0007**
Verbal 6-back, Post21-Pre	D-prime	**-0.688**	**0.0023**	**-0.698**	**0.0018**	0.05	0.4801
	RT	**-0.588**	**0.0131**	**-0.637**	**0.0060**	0.21	0.4169
	theta power	-0.217	0.4034	-0.395	0.1167	0.52	0.3016
	alpha power	0.157	0.5481	-0.210	0.4180	0.98	0.1635
	beta power	0.090	0.7312	-0.391	0.1207	1.33	0.0918
	Gamma power	0.275	0.2846	-0.466	0.0593	**2.08**	**0.0188**
	P300 GFP	0.283	0.2712	**-0.648**	**0.0049**	**2.81**	**0.0025**

Note: Bold indicates statistically significant results ($p < 0.05$).

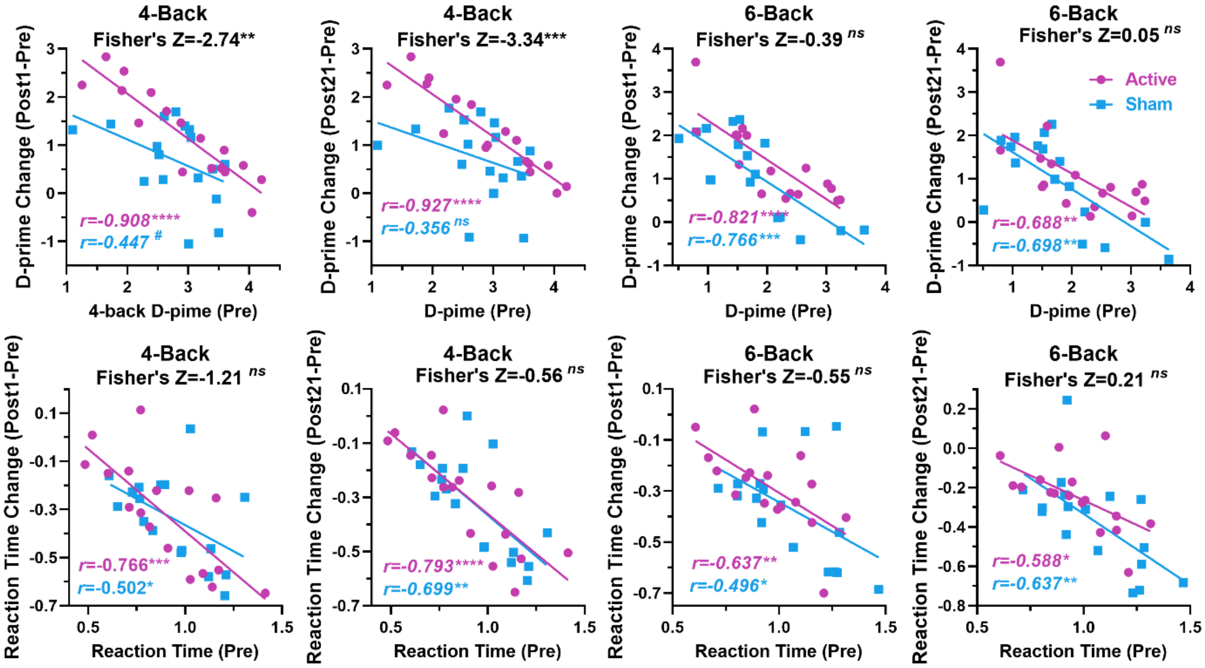

Supplementary Fig. 1 Behavioral performance prior to training correlates with changes in behavioral performance. P-values were determined by Fisher's Z-tests. (#: p<0.1; *: p<0.05; **: p<0.01; ***: p<0.001; ****: p<0.0001; *ns*: not significant)

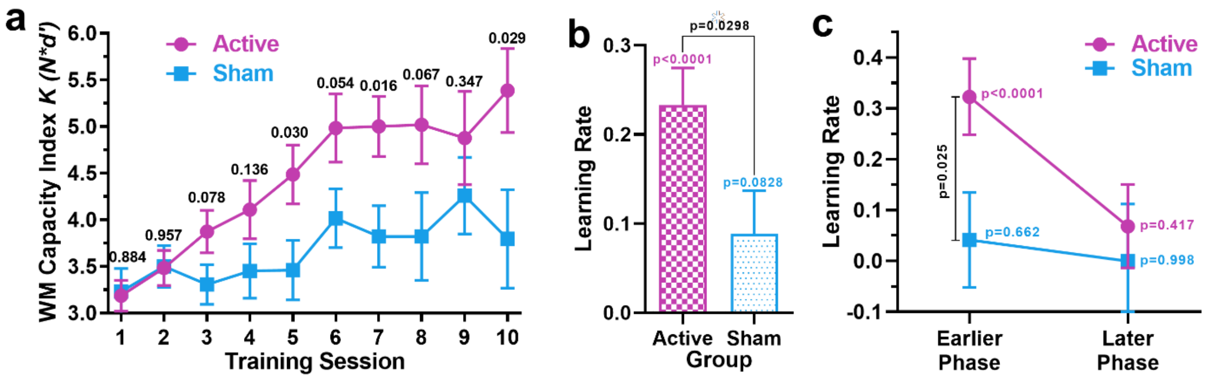

Supplementary Fig. 2. Learning curves and the corresponding learning rates during training. a Learning curves indicate the variations of the *K* index throughout the training course. The dash lines indicate the mean WM capacity indices. Significant main effects of group [F(1, 32)=4.284, p=0.047, η_p^2=0.118], training session [F(3.901, 124.821)=9.340, p<2e^{-6}, η_p^2=0.226, Greenhouse-Geisser correction] and group × training session interaction [F(3.901, 124.821)=2.478, p=0.049, η_p^2=0.072, Greenhouse-Geisser correction] have been revealed by two-way mixed ANOVA. Simple main effects analysis showed a significant effect of training session for active group [F(9, 144)=12.627, p<2e^{-14}, η_p^2=0.441] but not for sham group [F(9, 144)=1.596, p=0.122, η_p^2=0.091], suggesting significant training effect on the *K* value in active group. The numbers above the errorbars are the p values for main effect of group obtained from simple main effect analysis after the two-way MD-ANOVA. **b** Overall learning rates. Active group exhibited significantly higher learning rate than sham group as revealed by independent t-test [t(32)=2.275, p=0.0298, Cohen's d=0.780]. One-sample t-tests revealed significant learning rate (different from zero) for active group [mean=0.233, 95% CI=(0.145 0.321), t(16)=5.617, p<0.0001, Cohen's d=1.362] but not for sham group [mean=0.089, 95% CI=(-0.013 0.191), t(16)=1.851, p=0.0828, Cohen's d=0.449]. (**c**) Learning rates for different training phases. Two-way (group × training phase) MD-ANOVA revealed a significant main effect for training phase [F(1, 32)=5.159, p=0.030, η_p^2=0.139] but not for group [F(1, 32)=2.429, p=0.129, η_p^2=0.071] and group × training phase interaction [F(1, 32)=2.659, p=0.113, η_p^2=0.077)]. One-sample t-tests revealed significant divergence from zero only in earlier phase for active group (active group in earlier phase: mean=0.322, 95% CI=(0.163 0.481), t(16)=4.299, p<0.0001, Cohen's d=1.043; active group in later phase: mean=0.068, 95% CI=(-0.105 0.242), t(16)=0.834, p=0.417, Cohen's d=0.202; sham group in earlier phase: mean=0.041, 95% CI=(-0.156 0.239), t(16)=0.445, p=0.662, Cohen's d=0.108; sham group in later phase: mean=0.000, 95% CI=(-0.239 0.238), t(16)=-0.003, p=0.998, Cohen's d<0.001). Error bars depict SEM.

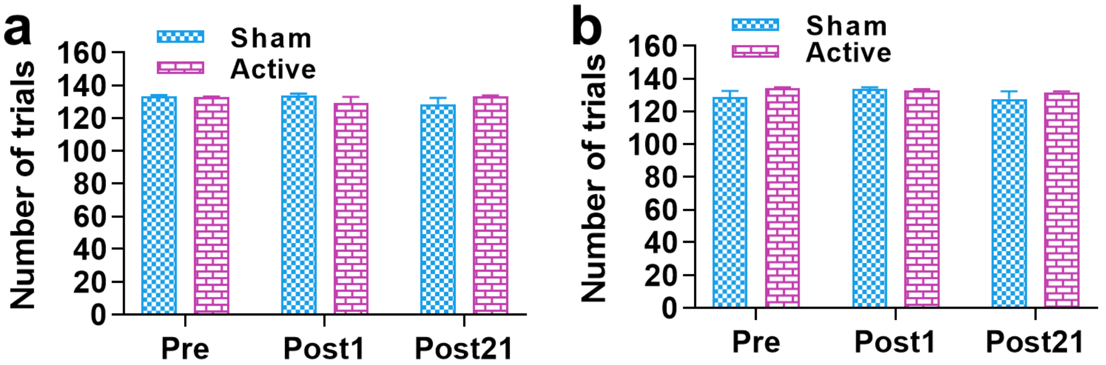

Supplementary Fig. 3. The number of EEG epochs included for analysis. Two-way (group × testing session) mixed ANOVA found no significant difference across groups and testing sessions. **a** Verbal 4-back [group: $F(1, 32) = 0.004$, $p=0.95$; testing session: $F(2, 64) = 0.446$, $p=0.597$; group × testing sessions interaction: $F(2, 64) = 2.033$, $p=0.139$]. **b** Verbal 6-back [group: $F(1, 32) = 2.184$, $p=0.149$; testing session: $F(2, 64) = 0.988$, $p=0.362$; group × testing sessions interaction: $F(2, 64) = 0.899$, $p=0.412$]. Error bars depict SEM.
