# Supplementary material for: Lasting enhancements in neural efficiency by multi-session transcranial direct current stimulation during working memory training
Source: NPJ Sci Learn. 2023 Nov 2;8:48. doi: 10.1038/s41539-023-00200-y (PMC10622507; doi:10.1038/s41539-023-00200-y)
Supplement: Supplementary file 2 — reporting-summary [file 41539_2023_200_MOESM2_ESM.pdf]

Corresponding author(s): Yufeng Ke

Last updated by author(s): Sep 9, 2023

Reporting Summary

Nature Portfolio wishes to improve the reproducibility of the work that we publish. This form provides structure for consistency and transparency in reporting. For further information on Nature Portfolio policies, see our Editorial Policies and the Editorial Policy Checklist.

Statistics

For all statistical analyses, confirm that the following items are present in the figure legend, table legend, main text, or Methods section.

n/a	Confirmed	
☐	☒	The exact sample size (n) for each experimental group/condition, given as a discrete number and unit of measurement
☐	☒	A statement on whether measurements were taken from distinct samples or whether the same sample was measured repeatedly
☐	☒	The statistical test(s) used AND whether they are one- or two-sided<br>*Only common tests should be described solely by name; describe more complex techniques in the Methods section.*
☐	☒	A description of all covariates tested
☐	☒	A description of any assumptions or corrections, such as tests of normality and adjustment for multiple comparisons
☐	☒	A full description of the statistical parameters including central tendency (e.g. means) or other basic estimates (e.g. regression coefficient) AND variation (e.g. standard deviation) or associated estimates of uncertainty (e.g. confidence intervals)
☐	☒	For null hypothesis testing, the test statistic (e.g. F, t, r) with confidence intervals, effect sizes, degrees of freedom and P value noted<br>*Give P values as exact values whenever suitable.*
☒	☐	For Bayesian analysis, information on the choice of priors and Markov chain Monte Carlo settings
☒	☐	For hierarchical and complex designs, identification of the appropriate level for tests and full reporting of outcomes
☐	☒	Estimates of effect sizes (e.g. Cohen's d, Pearson's r), indicating how they were calculated

Our web collection on statistics for biologists contains articles on many of the points above.

Software and code

Policy information about availability of computer code

Data collection	NeuroScan 4.5, PsychoPy
Data analysis	MATLAB ver 2014a, EEGLAB for MATLAB ver 15.0b, JASP ver 0.16.2, VassarStats website (http://vassarstats.net/rdiff.html)

For manuscripts utilizing custom algorithms or software that are central to the research but not yet described in published literature, software must be made available to editors and reviewers. We strongly encourage code deposition in a community repository (e.g. GitHub). See the Nature Portfolio guidelines for submitting code & software for further information.

Data

Policy information about availability of data

All manuscripts must include a data availability statement. This statement should provide the following information, where applicable:

- Accession codes, unique identifiers, or web links for publicly available datasets
- A description of any restrictions on data availability
- For clinical datasets or third party data, please ensure that the statement adheres to our policy

The sample datasets generated and analyzed during the current study are available from the corresponding author on reasonable request.

Research involving human participants, their data, or biological material

Policy information about studies with human participants or human data. See also policy information about sex, gender (identity/presentation), and sexual orientation and race, ethnicity and racism.

Reporting on sex and gender	Sex and gender are not considered in the study design. The gender information is provided by self-report.
Reporting on race, ethnicity, or other socially relevant groupings	Race, ethnicity, or other socially relevant groupings are not considered in the study design and are not provided.
Population characteristics	The participants were thirty-four graduate/undergraduate students (22.50±1.52 years old, including 18 males) .
Recruitment	Participants were university students.
Ethics oversight	the Ethics Committee of Tianjin University

Note that full information on the approval of the study protocol must also be provided in the manuscript.

Field-specific reporting

Please select the one below that is the best fit for your research. If you are not sure, read the appropriate sections before making your selection.

☒ Life sciences ☐ Behavioural & social sciences ☐ Ecological, evolutionary & environmental sciences

For a reference copy of the document with all sections, see nature.com/documents/nr-reporting-summary-flat.pdf

Life sciences study design

All studies must disclose on these points even when the disclosure is negative.

Sample size	Data were sampled by convenience on campus, whose size was determined in light of existing studies. Certainly, the authors agree that increases sample size of is very helpful to add power to this study. However, thirty-four subjects for 10 days tDCS+working memory training studies with follow-up EEG recording and task-related EEG analysis are still not a small sample size in the literatures.
Data exclusions	EEG epochs with obvious noise were manually removed. The average numbers of EEG epochs included in following analysis were around 130 with no significant difference across groups and testing sessions for all the tasks
Replication	The reproducibility lies on the fact that the observations from a small size of pilot study was largely consistent with the final experimental finding.
Randomization	The participants were randomized into two groups in this study.
Blinding	The data analyzers were single-blinded to the grouping. The data collector was not possible to be blinded with the grouping for the experimental design.

Reporting for specific materials, systems and methods

We require information from authors about some types of materials, experimental systems and methods used in many studies. Here, indicate whether each material, system or method listed is relevant to your study. If you are not sure if a list item applies to your research, read the appropriate section before selecting a response.

Materials & experimental systems

n/a	Involved in the study	
☒	☐	Antibodies
☒	☐	Eukaryotic cell lines
☒	☐	Palaeontology and archaeology
☒	☐	Animals and other organisms
☒	☐	Clinical data
☒	☐	Dual use research of concern
☒	☐	Plants

Methods

n/a	Involved in the study	
☒	☐	ChIP-seq
☒	☐	Flow cytometry
☒	☐	MRI-based neuroimaging
